# Activation of arcuate nucleus glucagon-like peptide-1 receptor-expressing neurons suppresses food intake

**DOI:** 10.1186/s13578-022-00914-3

**Published:** 2022-10-29

**Authors:** Ishnoor Singh, Le Wang, Baijuan Xia, Ji Liu, Azeddine Tahiri, Abdelfattah El Ouaamari, Michael B. Wheeler, Zhiping P. Pang

**Affiliations:** 1 The Child Health Institute of New Jersey, Robert Wood Johnson Medical School, Rutgers, The State University of New Jersey, New Brunswick, NJ 08901 USA; 2 Department of Physiology, University of Toronto, 1 King’s College Circle, Toronto, ON M5S 1A8 Canada; 3 School of Basic Medical Sciences, Guizhou Medical University, Guiyang, Guizhou, 550025 China; 4 National Engineering Laboratory for Brain-Inspired Intelligence Technology and Application, School of Information Science and Technology, University of Science and Technology of China, Hefei, 230026 Anhui China; 5 Department of Medicine, Division of Endocrinology, Metabolism and Nutrition, Robert Wood Johnson Medical School, Rutgers, The State University of New Jersey, New Brunswick, NJ 08901 USA; 6 Metabolism Research Group, Division of Advanced Diagnostics, Toronto, ON Canada; 7 Department of Neuroscience and Cell Biology, Rutgers, The State University of New Jersey, New Brunswick, NJ 08901 USA; 8 Department of Pediatrics, Robert Wood Johnson Medical School, Rutgers, The State University of New Jersey, New Brunswick, NJ 08901 USA

**Keywords:** Glucagon-like peptide-1, Hypothalamus, Pro-opiomelanocortin, Exendin-4, Chemogenetics, Glucose tolerance, Feeding

## Abstract

**Background:**

Central nervous system (CNS) control of metabolism plays a pivotal role in maintaining energy balance. In the brain, Glucagon-like peptide 1 (GLP-1), encoded by the proglucagon ‘*Gcg*’* gene*, produced in a distinct population of neurons in the nucleus tractus solitarius (NTS), has been shown to regulate feeding behavior leading to the suppression of appetite. However, neuronal networks that mediate endogenous GLP-1 action in the CNS on feeding and energy balance are not well understood.

**Results:**

We analyzed the distribution of GLP-1R-expressing neurons and axonal projections of NTS GLP-1-producing neurons in the mouse brain. GLP-1R neurons were found to be broadly distributed in the brain and specific forebrain regions, particularly the hypothalamus, including the arcuate nucleus of the hypothalamus (ARC), a brain region known to regulate energy homeostasis and feeding behavior, that receives dense NTS^Gcg^ neuronal projections. The impact of GLP-1 signaling in the ARC GLP-1R-expressing neurons and the impact of activation of ARC GLP-1R on food intake was examined. Application of GLP-1R specific agonist Exendin-4 (Exn-4) enhanced a proportion of the ARC GLP-1R-expressing neurons and pro-opiomelanocortin (POMC) neuronal action potential firing rates. Chemogenetic activation of the ARC GLP-1R neurons by using Cre-dependent hM3Dq AAV in the *GLP-1R-ires-Cre* mice, established that acute activation of the ARC GLP-1R neurons significantly suppressed food intake but did not have a strong impact on glucose homeostasis.

**Conclusions:**

These results highlight the importance of central GLP-1 signaling in the ARC that express GLP-1R that upon activation, regulate feeding behavior.

**Supplementary Information:**

The online version contains supplementary material available at 10.1186/s13578-022-00914-3.

## Introduction

Glucagon-like peptide 1(GLP-1), an incretin hormone encoded by the proglucagon *Gcg* gene, is produced by intestinal enteroendocrine L cells and a subpopulation of hindbrain nucleus tractus solitarius (NTS) neurons in both rodents and humans (for review, see [1]). GLP-1 actions are mediated by the GLP-1 receptor (GLP-1R), a class B subfamily G-protein-couple-receptor, widely expressed in central and peripheral nervous systems [2–4]. There is now ample evidence suggesting that the endogenous GLP-1 signaling works on both peripheral organs and the brain to regulate energy homeostasis, including but not exclusively through glucose metabolism and food intake [5]. GLP-1R agonists are now widely used therapeutics to treat type 2 diabetes and more recently obesity [5, 6]. Studies in animals have demonstrated that central GLP-1-producing NTS^Gcg^ neurons project to various regions in the forebrain [7]. Still, the physiological role of endogenous GLP-1 signaling in the control of food intake and body weight remains enigmatic [5].

Despite the clear indication that acute intracerebroventricular injections of GLP-1R antagonist exendin-9 in rats increases food intake [8], various genetic perturbation of the GLP-1Rs, including a knockout of GLP-1R causes little effect on food intake or body weight [9–11]. While inhibition of GLP-1 expressing neurons in the brainstem increases feeding, food intake and meal size, and activation of these neurons suppress feeding; postnatal ablation of GLP-1 neurons does not impact food intake, meal size, energy expenditure, or body weight [12]. Although Burmeister et al. used multiple genetic approaches, including *Nkx2-Cre, Pomc-Cre, Sim1-Cre*, or *nuclear receptor 5A1 (Nr5a)-Cre,* to cross with *floxed GLP-1R* (i.e., *GLP-1R*^*f/f*^) to broadly or selectively ablate the GLP-1R in mouse hypothalamus, these mice failed to show changes in the body weight [13, 14]. However, knockdown of GLP-1R within the lateral hypothalamus by RNAi in adult rats caused a significant increase in food intake, body weight, and fat mass [15]. Consistent with this notion, knockdown of GLP-1R in the dorsomedial hypothalamus increased food intake, decreased energy expenditure, and increased body weight over weeks of observation [16]. Also, the knockdown of the *Gcg* gene in the NTS increased food intake and body weight in high-fat-fed rats [17]. In addition, chemogenetic stimulation of *Gcg* neurons suppressed feeding when fed or fasted animals were fed chow or a high-fat diet [18]. On the other hand, acute chemogenetic inhibition of Gcg neurons did not increase ad libitum feeding but increased refeeding after fasting and blocked stress-induced hypophagia [19]. In our previous study, we found that NTS GLP-1 neurons project to the paraventricular hypothalamic nucleus (PVN) and enhance glutamatergic synaptic transmission, which is sufficient to suppress food intake and specific ablation of PVN GLP-1R causes overeating and obesity [20]. Nevertheless, the CNS GLP-1 signaling in regulating feeding behavior does not seem to be activated by peripherally secreted endogenous GLP-1 and, therefore, may be distinct from the peripheral GLP-1 system. Central and peripheral GLP-1 systems have been presumed to independently suppress the feeding [12]. These results highlight the complexity of GLP-1 regulation of energy homeostasis, but the cellular and circuit mechanisms mediating endogenous GLP-1 action in the CNS are still poorly understood. This is partly due to the presence of diverse neuronal subtypes and complex central neuronal connectivity in the brain.

The NTS^Gcg^ neurons send robust projections to multiple brain regions [12, 21, 22], especially to the mediobasal hypothalamic regions; PVN, lateral hypothalamus (LH), ventromedial nucleus of the hypothalamus (VMH) and arcuate nucleus of the hypothalamus (ARC) [7]. The ARC is an evolutionarily conserved brain region with essential roles in regulating the energy homeostasis [23]. The various ARC functions are supported by specialized neuronal subtypes, including two well-characterized neuronal populations that control feeding and insulin sensitivity: Agouti-related protein (AgRP) and Proopiomelanocortin (POMC) neurons [24–26]. However, a recent study that utilized the single-cell RNA-seq technology provided further evidence regarding the heterogeneous POMC cell populations within the ARC and revealed that the chemogenetic activation of POMC GLP-1R-expressing neurons could remarkably suppress feeding while activation of POMC Leptin receptor-expressing neurons cannot [27]. These results indicate that GLP-1 signaling plays an essential role within the ARC neurons.

Synaptic transmission mediates information flow in the brain to control behavior, including feeding and food intake [28]. Previous studies have shown that GLP-1R signaling may facilitate excitatory presynaptic release [29, 30] and increase presynaptic vesicle release probability in the mesolimbic dopamine system [31]. Our group has shown that GLP-1R activation suppresses excitatory synaptic strength in the mesolimbic pathway [32]. However, in the PVN, GLP-1R activation enhances AMPA receptor trafficking and the excitatory postsynaptic strength [20]. It also has been shown that the GLP-1R agonist, semaglutide/liraglutide, directly activates POMC neurons and indirectly inhibits NPY/AGRP neurons in the ARC [33, 34]. These results suggest that GLP-1 might play a distinctive role in different cell types and brain regions through the distinct mechanism to regulate neuronal functions. Nevertheless, the mechanistic regulation of GLP-1 signaling in modulating ARC neurons is not yet apparent.

To elucidate the involvement of GLP-1 signaling in the ARC neurons in regulating feeding behavior, we used *GLP-1R-ires-Cre* knock-in mice [35] and *Gcg*-Cre recombinase transgenic mice [18], which are unique genetic models for specifically investigating the functions of GLP-1R in the brain and the *Gcg* (proglucagon) GLP-1 releasing neurons located in the hindbrain, respectively. First, we systematically characterized the anatomy of the GLP-1R neuronal distribution in the whole brain using the *GLP-1R-ires-Cre* mice crossed with Ai14 reporter mice [36]. Next, we evaluated the GLP-1 neuronal (NTS^Gcg^) projections in the mouse brain. Our data show that GLP-1R-expressing neurons are widely distributed in multiple brain regions, including mediobasal hypothalamic areas, such as the PVN, ARC, and VMH. Meanwhile, these brain regions also receive dense NTS^Gcg^ neuronal inputs. Since the ARC is an established significant target of the CNS GLP-1 system [37, 38], we focused on elucidating the ARC GLP-1 signaling in regulating synaptic transmission as well as feeding behavior. Our whole-cell patch-clamp recording analysis shows that Exn-4 enhances the action potential firing frequency in a proportion of ARC GLP-1R-expressing neurons and POMC neurons. Finally, acute chemogenetic activation of ARC GLP-1R neurons suppresses feeding dramatically but had no strong impact on glucose tolerance.

## Results

### Distributions of GLP-1R neurons in the mouse brain

**Fig. 1:**
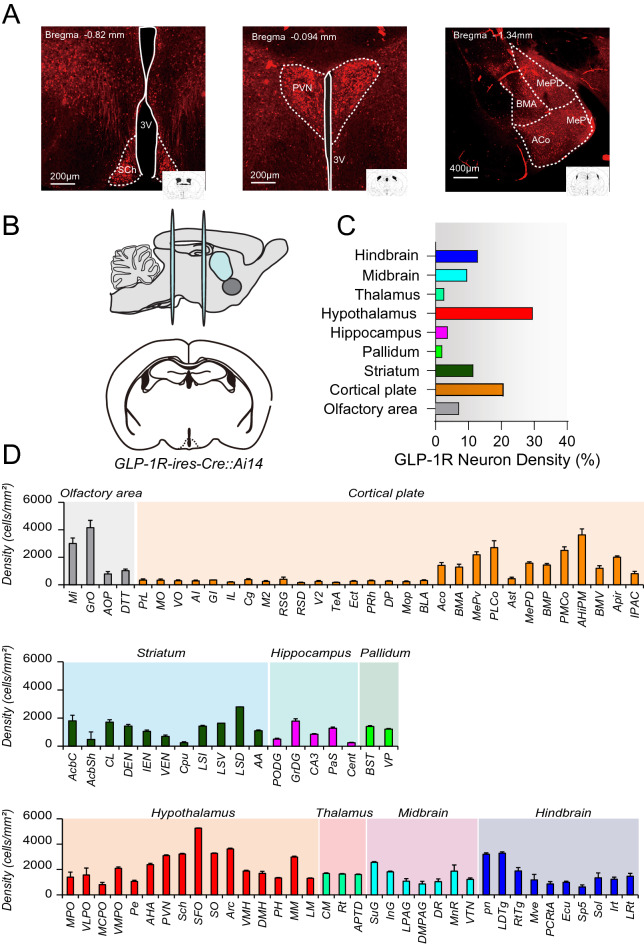
Quantification of GLP-1 receptor distribution in the whole brain. **A** Representative images of *GLP-1R-ires-cre* mouse crossed with Ai14 tdTomato *(LSL-TdTomato)* reporter mice in Suprachiasmatic nucleus (Sch), Paraventricular nucleus of the hypothalamus (PVN), Basomedial amygdaloid nucleus (BMA), anterior cortical amygdaloid area (ACo), medial amygdaloid nucleus (MeP). **B** Whole brain slice preparation of *GLP-1R-ires-Cre::Ai14 tdTomato*. **C** Percentage (%) of GLP-1R neuronal density in the brain. **D** Quantification of the total number of GLP-1R neurons area (density) in the brain. Data are presented as mean ± standard error of the mean (SEM), [n = 3]

To comprehensively investigate the whole-brain distribution of GLP-1R-expressing neurons, GLP-1R positive neurons were genetically labeled using *GLP-1R-ires-Cre* mice crossed to the tdTomato fluorescent reporter line Ai14 [36]. Consistent with previous reports, we found GLP-1R expression neurons, based on the expression of tdTomato (Fig. 1A). We systematically analyzed the cell number and cell density across the whole brain (Fig. 1B, C). In three animals, a total of 101,618 GLP-1R-expressing (tdTomato) neurons were counted from a series of 50 μm sections (every 6 sections). The majority of GLP-1R neurons were found in the hypothalamus (~ 29.59%), cortical regions (~ 20.71%), striatum (~ 11.54%), and midbrain (~ 9.6%). Within the hypothalamic region (Fig. 1D), dense populations of GLP-1R neurons were enriched in the subconical organ (SFO), ARC, supraoptic nucleus (SO), the suprachiasmatic nucleus (Sch), and paraventricular hypothalamic nucleus (PVN). Notably, the ARC region possesses a high abundance of GLP-1R neurons, consistent with a previous report on GLP-1R expression using a super-resolution fluorescent probe in the brain [39]. In addition, a high density of GLP-1R neurons are also found in the cortex, striatum [21], thalamus, and midbrain regions indicating that GLP-1 signaling also has essential functions in both cortical and subcortical neuronal networks (Fig. 1C; Additional file 1: Fig. S1 and Additional file 2: Fig. S2).

### Axonal projection patterns of NTS^Gcg^ neurons

**Fig. 2:**
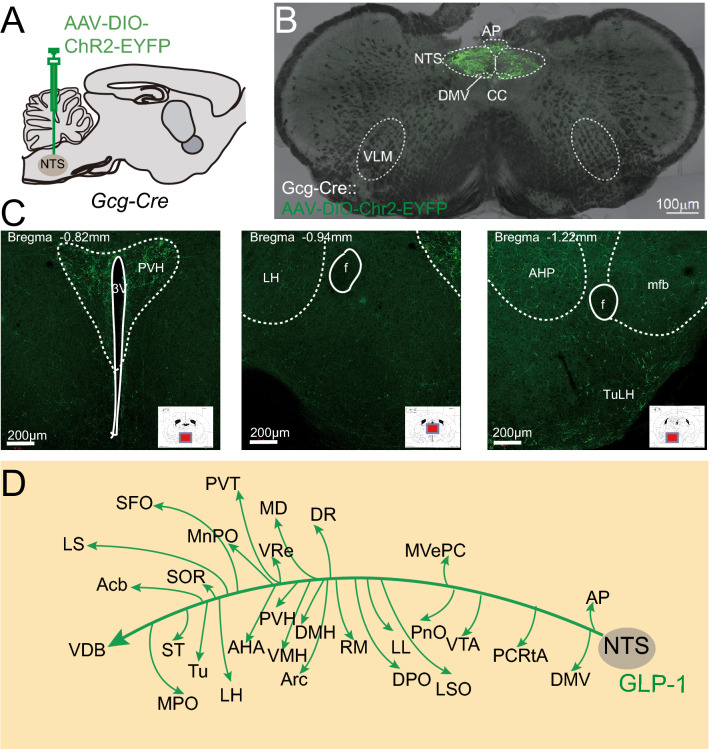
Axonal projection patterns of NTS Gcg endogenous GLP-1 neurons. **A** Gcg-Cre mouse injected with AAV-DIO-Chr2-EYFP in the NTS. **B** Representative image of the injection site in the Gcg-Cre mouse injected with AAV-DIO-Chr2-EYFP in the NTS. **C** Representative images of the axon terminals in the brain regions of Gcg-Cre mouse injected with AAV-DIO-ChR2-EYFP. **D** Identified brain regions with axon terminals from NTS^Gcg^ (GLP-1) neurons. A map representing the projection patterns of the GLP-1 neurons in the brain (n = 3)

A previous immunohistochemical study using custom-made GLP-1 monoclonal antibodies examined the distribution of GLP-1 expressing nerve terminals in the rat forebrain regions [7], but provided limited information about the overall GLP-1 neuronal projections in the mouse brain. To evaluate the NTS^Gcg^ neuronal projections in the mouse brain, we injected AAV-DIO-ChR2-EYFP into the NTS region of the Gcg-Cre mice [18] (Fig. 2A and B). The morphological analysis of the axonal terminals revealed dense GLP-1 neuronal terminals in the hypothalamus, especially in the PVN, DMH, ARC, and the medial vestibular parvicellular subfields nucleus (MvePC) (Fig. 2C and D). We also found dense projections in the median preoptic nucleus (MnPO), medial dorsal nucleus (MD), dorsal raphe nucleus (DR), and pontine reticular nucleus, oral part (PnO) (Additional file 3: Fig. S3 and Additional file 4: Fig. S4). These projections ascertain the prime brain regions involved in the GLP-1 signaling which regulate brain and body functions but are not limited to food intake, body weight, glucose metabolism, learning, and memory.

### Modulation of neuronal activity in ARC by GLP-1 signaling

**Fig. 3:**
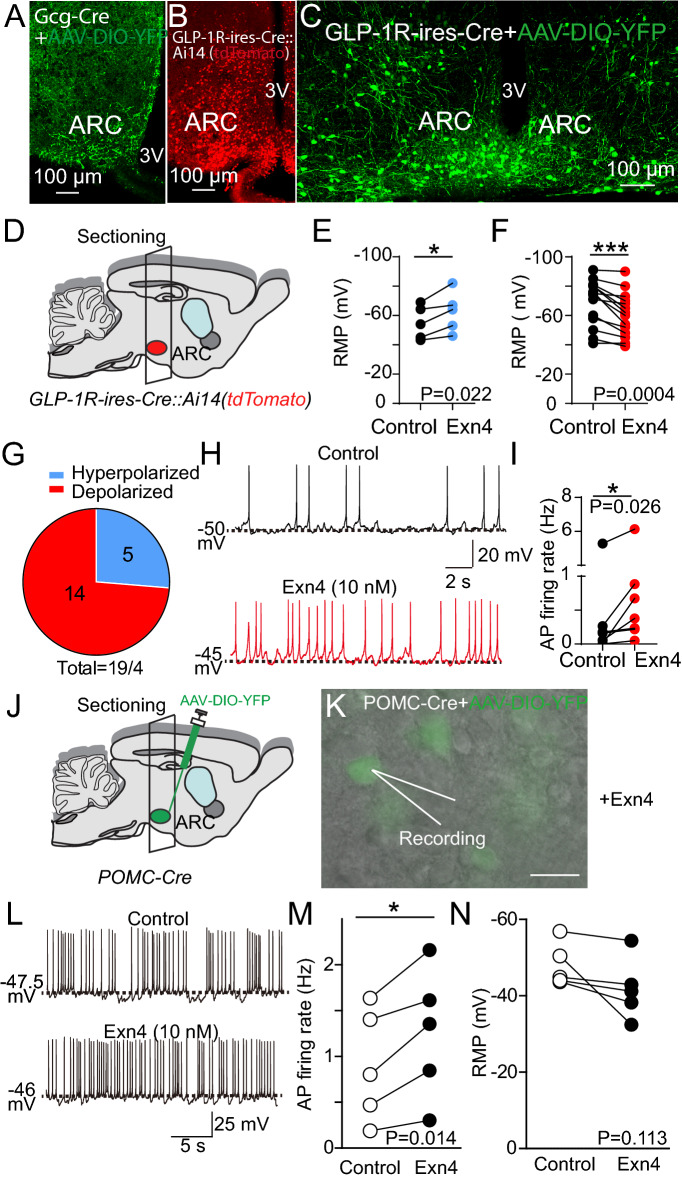
Expression of GLP-1R in the ARC and the effects of Exn4 on ARC neurons. **A** GLP-1 neuronal axonal projections in the ARC region. AAV-DIO-Chr2-EYFP was injected into the NTS region of the *Gcg-Cre* mouse. **B** GLP-1R-expressing neurons in the ARC region revealed by tdTomato fluorescence in *GLP-1R-ires-Cre::Ai14 (LSL-TdTomato)* mice. **C** Expression of GLP-1R in the ARC using a conditional viral-mediated approach. AAV-DIO-eGFP was injected into the ARC region of the *GLP-1R-ires-Cre* mouse. **D** Experimental paradigm for recordings from GLP-1R-expressing neurons labeled with tdTomato in *GLP-1R-ires-cre::*Ai14 tdTomato *(Lox-Stop-Lox-TdTomato)* reporter mice. **E** and **F** The resting membrane potential (RMP) of ARC GLP-1R-expressing neurons in the absence or presence of Exn4. Paired t-test, *p < 0.05, ***p < 0.001. **G** Pie chart of the proportion of ARC GLP-1R-expressing neurons showing RMP changes in the presence of Exn-4. **H** Represent traces of membrane potentials with action potentials (APs) in the ARC GLP-1R neurons with or without Exn4. **I** Pooled data. Recordings were conducted in 4 animals. Paired t-test, *p < 0.05. **J** and **K** Experimental paradigm for recordings in POMC-neurons. *POMC-Cre* mice were injected with AAV-DIO-eYFP in the ARC. **L** Representative traces of APs in POMC neurons in the absence or presence of Exn4. **M** and **N** Pooled data. Data are presented as mean + S.E.M., n = 5/2 cells/animals. Paired t-test, *p < 0.05

The ARC region of the hypothalamus receives strong innervation from the NTS GLP-1 nerve terminals (Fig. 3A) and notes the abundance of the GLP-1R expressed in the ARC neurons (Fig. 3B and C). We next investigated the cellular impacts of GLP-1 signaling in the ARC neurons. We labeled ARC GLP-1R neurons by crossing GLP-1R-ires-Cre with Ai14 reporter mice (Fig. 3D). Whole-cell patch-clamp recording from the tdTomato-expressing GLP-1R neurons revealed the application of Exendin 4 (Exn4), a specific agonist of GLP-1R [40], regulates ARC neuronal firings. Our data suggested that GLP-1R signaling produces diverse cellular responses in the ARC GLP-1R-expressing neurons: ~ 75% of GLP-1R neurons showed depolarization (Fig. 3E), while the rest showed hyperpolarization (Fig. 3F and G). Moreover, Exn4 significantly increased the firing rates of neurons showing spontaneous action potentials (Fig. 3H and I). Next, we investigated the impact of Exn4 on one of the major neuronal types, POMC neurons in ARC. We labeled POMC neurons using a POMC-Cre mice [41] with Cre-dependent expression of GFP (AAV-DIO-eGFP) (Fig. 3J and K). Whole-cell patch-clamp recording from the POMC neurons revealed that Exn4 increased the AP firing of POMC neurons with a slight depolarization (Fig. 3L–N). The GLP-1R neurons recording data suggested a heterogenous regulation by GLP-1R mediated signaling, possibly due to the diverse cell types in the ARC.

### Activation of ARC GLP-1R neurons does not regulate blood glucose and insulin secretion

**Fig. 4:**
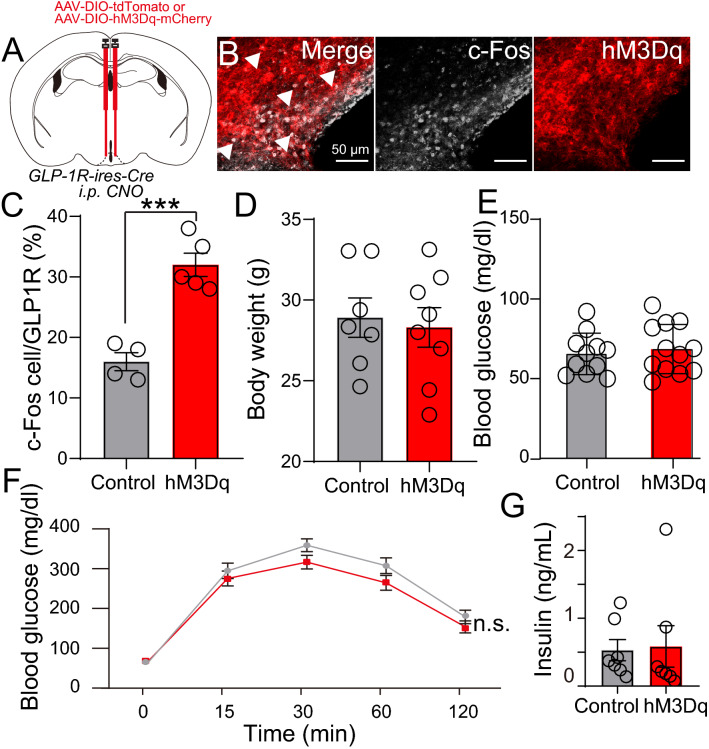
Acute activation response of ARC GLP-1R neurons on blood glucose. **A** Experimental paradigm for virus delivery and chemogenetic stimulation of the GLP-1R neurons in ARC. **B** Representative image of hM3Dq (red) and c-Fos (white) staining in the *GLP-1R*-ires-Cre ARC region. **C** Quantification of c-Fos and GLP-1R co-localized neurons in the ARC after chemogenetic activation (CNO) control n = 4, hM3Dq n = 5. **D** Average bodyweight of the control and hM3Dq groups after overnight fasting (12 h) control n = 7, hM3Dq n = 8. **E** Fasting glucose levels control n = 11, hM3Dq n = 12. **F** IP GTT glucose levels upon ARC GLP-1R neuronal activation, p-value = 0.0633 control n = 11, hM3Dq n = 12. **G** Insulin levels after 1 h ARC GLP-1R neuron activation via CNO delivery. Data are presented as mean ± standard error of the mean (SEM). Student’s t-test (Bar-graph); *p < 0.05; **p < 0.01, ***p < 0.001. Two-way Anova, with Geisser-Greenhouse correction

Using pharmacological manipulations, it has been suggested that the ARC GLP-1 receptor-mediated signaling regulates glucose homeostasis, not food intake [2]. On the other hand, the role of central GLP-1 in regulating blood glucose and appetite has gained attention and has been reported in numerous studies. Hence, we next asked if chemogenetically activating the ARC GLP-1R neurons will affect glucose metabolism. To this end, we used Designer Receptor Exclusively Activated by Designer Drugs (DREADDs) [42] technology to activate ARC GLP-1R-expressing neurons. We injected AAV-DIO-hM3Dq-DREADD and AAV-FLEX-tdTomato (Control) into the ARC of the GLP-1-ires-Cre mice. 4 weeks post-injection, we administered DREADDs ligand clozapine-N-oxide (CNO, 1 mg/kg body weight) via intraperitoneal (i.p.) route. We confirmed that chemogenetic activation of GLP-1R neurons increased the expression of the c-Fos protein, an early-intermediate marker of neuronal activity (Fig. 4A–C). To evaluate the impact of the GLP-1R neurons mediated signaling on glucose metabolism, we performed a glucose tolerance test (GTT) and measured plasma levels of insulin. Thirty minutes after CNO injection, 1 g/kg glucose was administered *i.p.,* however, the blood glucose levels show no difference (Fig. 4D–F). Similarly, no plasma insulin level changes were found at 1 h after CNO injection (Fig. 4G). Collectively, these data suggest that under the conditions studied, the ARC GLP-1R neurons in vivo does not mediate glucose tolerance and insulin secretions upon direct acute activation.

### Activation of ARC GLP-1R neurons suppresses food intake behaviors

**Fig. 5:**
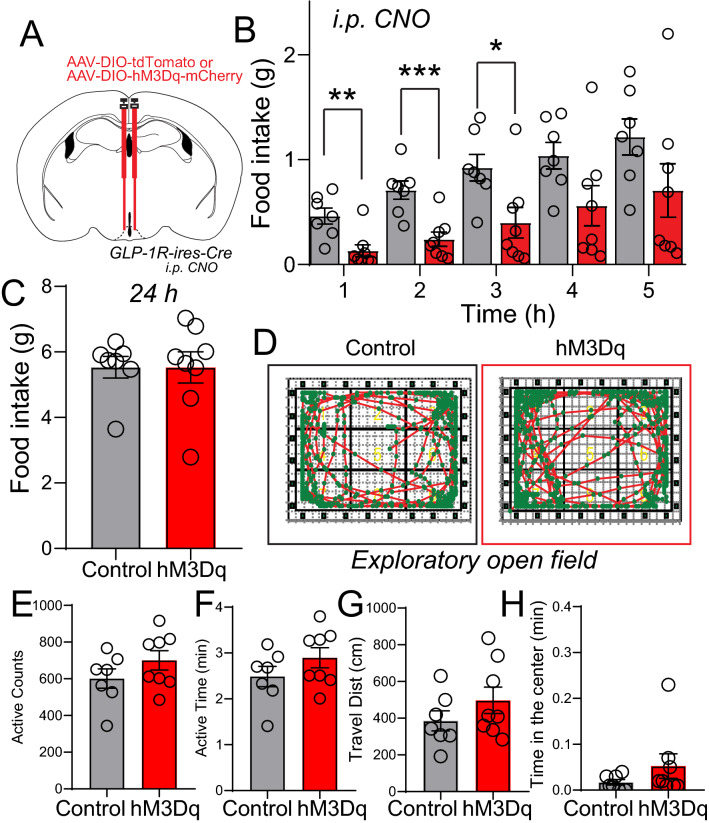
Acute activation response of ARC GLP-1R neuron on feeding behavior. **A** Experimental paradigm for virus delivery and chemogenetic stimulation of the GLP-1R neurons in ARC. **B** Food intake consumption upon activation of the GLP-1R neurons in ARC (0.5 g food fed overnight), p value = 0.0261*, control n = 7, hM3Dq n = 8. **C** Food intake consumption after 24 h CNO injection. **D** In an exploratory open field test, the dependent locomotor activity of control and hM3Dq mice after CNO delivery, measuring various parameters. **E** Active count. **F** Total active time (min). **G** Total traveled distance (cm). **H** Time spent in the cage center (min). Data are presented as mean ± standard error of the mean (SEM). Student’s t-test; *p < 0.05; **p < 0.01, ***p < 0.001. Two-way Anova, with Geisser-Greenhouse correction

We next assessed the impact of activation of the ARC GLP-1R neurons on feeding behavior. We hypothesized that the activation of the GLP-1R neurons in the ARC might affect food intake. Animals (GLP-1R-ires-Cre mice with Cre-dependent expression of hM3Dq or tdTomato AAV vectors injected in the ARC) in this cohort were singly housed for 3 days, fasted overnight, and GLP-1R neurons were activated by *i.p.* CNO (Fig. 5A). Acute activation of ARC GLP-1R neurons led to significant decreases in food intake compared to control mice expressing mCherry alone (Fig. 5B). The effect of the DREADD ligand seemed to dissipate after 12 h; the activation had little impact on total food intake within 24 h after the application of CNO (Fig. 5C). This possibly could be due to the relatively short half-life of the DREADD ligand CNO [43].

Because the behavioral state can influence feeding, we examined whether the inhibition of food intake was due to the presence of anxiety [44]. Mice from the previous experiments were placed in an open-field arena 30 min after injection of CNO. No differences were observed between mCherry and hM3Dq-expressing mice in the time spent in the center of the open field, providing evidence that activation of the ARC GLP-1R neurons does not cause overt anxiety (Fig. 5D–H). Overall, the data show that activation of the GLP-1R neurons in mice leads to repetitive behaviors that are not due to increased anxiety levels but remarkably suppressed feeding behavior.

## Discussion

In this study, using viral-mediated labeling and genetically modified animal models, we surveyed the distribution of GLP-1R neurons and the overall projection patterns of the GLP-1-producing neurons in the mouse brain. In brain slices, activation of GLP-1R with specific agonist Exn-4 enhanced the firing rate of a proportion of the ARC neurons including the POMC-expressing neurons. We then found that activation of the ARC GLP-1R neurons suppressed food intake without a noticeable impact on glucose metabolism or causing any stress-related responses. This result highlights the importance of the central function of GLP-1 signaling within the ARC in regulating metabolism.

A widespread distribution of the GLP-1R neurons in the mouse brain was located, including the hypothalamus, cortical regions, striatum, and midbrain. It is interesting to note that NTS^GCG^ neurons do not project to all these areas with the same density nor are equivalent to the distribution of the GLP-1R density in the brain (Fig. 2). For example, our previous research indicates that in the PVN [20] and DMH [45], the high density of GLP-1R-expressing neurons is consistent with a high density of GLP-1 fiber terminals, and the GLP-1 neurons from the NTS form synapses onto the postsynaptic neurons. On the other hand, although high-level of GLP-1R-expressing neurons are found in the hippocampus [46], and primary cortex [47], very few GLP-1 neuronal fiber projections were found (Fig. 1). The mismatched projections/receptors distribution is not unique to GLP-1 and has been described in other peptidergic or nonpeptidergic transmitter systems [48, 49]. This suggests that GLP-1 release may affect postsynaptic neuronal functionality and non-synaptic volume transmission via synaptic release or diffusion through the tissue to their target sites [50]. Another possibility is that circumventricular organs are located outside the blood–brain barrier, suggesting that GLP-1 of peripheral origin could be the ligand of these receptors. Indeed, the binding of peripherally administered GLP-1 to the SFO and the area postrema (AP) has been demonstrated by [51]. Given that some GLP-1 analogs are FDA-approved drugs to treat obesity [5], they may function via these widespread GLP-1R-expressing neurons. Interestingly, the ARC has a high-density distribution of GLP-1 nerve terminals as well as high density of GLP-1R neurons. The ARC is in close proximity to the third ventricle and with incomplete brain blood barriers [52], which makes it the prime target of both circulating peripheral GLP-1 (or pharmacological GLP-1 analogs) and central GLP-1 regulations. For example, it has been shown that peripheral application of fluorescently labeled liraglutide binds to neurons (presumably GLP-1R-expressing neurons) within the ARC [33].

Ample studies have demonstrated that various ARC functions are supported by specialized neuronal subtypes [22, 24–26]. For example, ARC POMC neurons are activated by energy surfeit, and their activations suppress feeding and promote weight loss [53]; while AgRP neuronal activations promote feeding [24, 54, 55]. Further, peripherally released ghrelin hormone suppresses food intake via ARC [56]. ARC POMC neurons mediate serotonin signaling regulation of the energy homeostasis [57]. A recent elegant study using mouse genetics revealed the profound and redundant function of ARC neurons in the obesity development [58]. ARC POMC neurons are known to be regulated by GLP-1 signaling [27, 33, 59]. It has been reported that POMC neurons express GLP-1R but not AgRP neurons [23]. Notably, a recent study defined distinct POMC neurons expressing leptin receptor and GLP-1R exhibiting a distinct anatomical distribution and electrophysiological properties [27], suggesting further investigation of the heterogeneous function mediated by these diverse cell types in the ARC is required. To that fact, when GLP-1R agonist, Exn4 is applied to this region, it enhances the ARC POMC neuronal spontaneous action potential while decreasing the trend membrane potential (Fig. 3L–N), which is consistent with the previous findings of GLP-1/Liraglutide modulating ARC POMC/CART neurons [33, 34, 60]. Meanwhile, Exn4 also showed a suppression of non-POMC neurons’ spontaneous action potential firing frequency, suggesting an enhanced inhibition, which is consistent with the recent findings in the ARC NPY neurons [34]. This data suggests that the GLP-1 can modulate cell-type-specific POMC neuronal activity within the ARC region.

Chemogenetic activation of the NTS^Gcg^ neurons in the brainstem promotes glucose homeostasis [22]. GLP-1R agonist CNS infusion has shown that the arcuate GLP-1 receptors are a crucial component of the GLP-1 system for improving glucose homeostasis by regulating insulin secretion and glucose production without affecting food intake [61]. Meanwhile, a previous study has demonstrated that the ARC AgRP neurons in mice rapidly coordinate hunger states with glucose homeostasis by stimulating the expression of muscle-related genes in brown adipose tissue [26]. These data strongly suggest that GLP-1 signaling in the ARC regulates glucose homeostasis. However, our study found that acute GLP-1R neuronal activation causes insignificant changes in blood glucose clearance and insulin secretion (Fig. 4). These data suggest that the blood glucose regulatory function of ARC GLP-1 signaling may not be via the acute activation of ARC GLP-1R neurons; or GLP-1 might modulate GLP-1R expressing presynaptic terminal projected to the ARC region (e.g., PVN, DMH). Interestingly, our recent study revealed that GLP-1R neurons in the dorsomedial hypothalamus are glucose-sensing neurons and are involved in regulating glucose metabolism [45]. Further investigations are needed to address the detailed mechanism of the GLP-1 signaling in regulating glucose metabolism and the involvement of postsynaptic GLP-1R in the ARC.

Despite the report that ARC GLP-1 signaling regulates glucose metabolism but not feeding behavior [61], it has also been shown that GLP-1R agonist liraglutide infusion in the ARC induces weight loss in rodents [33]. A recent study has demonstrated that the chemogenetic activation of POMC^Glp1r+^ neurons remarkably suppresses the feeding behavior [27]. Consistent with this notion, we found that the chemogenetic activation of the ARC GLP-1R neurons remarkably reduces food intake upon hM3Dq ligand injections (i.p., CNO). This suggests that acute activation of the GLP-1R neurons in the ARC robustly reduces food intake and might be a key player in regulating energy homeostasis (Fig. 5). While our data do not test GLP-1 signaling in the ARC directly, we show that the ARC POMC neurons are activated by GLP-1 signaling (Fig. 3), and the activation of the ARC GLP-1R neurons significantly suppresses feeding (Fig. 5). Here, we want to note that the chemogenetic activation of the ARC GLP-1R neurons is not equivalent to the GLP-1 signaling activation. These data suggest that the GLP-1R neuronal circuitry in the brain, including the ARC, plays a pivotal role in regulating energy homeostasis.

## Conclusion

Taken together, these experiments revisit the distribution patterns of GLP-1R-expressing neurons and NTS^Gcg^ neuron projections in the mouse brain. Our study suggests that the GLP-1R mediated signaling regulates postsynaptic ARC neuronal excitability and activation of these GLP-1R neurons modulate energy homeostasis by regulation of appetite.

## Materials and methods

### Animals

All studies and procedures involving mice were approved by the Rutgers University Institutional Animal Care and Use Committee (IACUC) and by the National Institute of Health (NIH) guidelines. The animals used in this study were 5–7 weeks old, housed, and bred in the Child Health Institute of New Jersey facility. We used cell-type-specific expression of Cre-recombinase mouse lines; GLP-1R-ires-Cre [35], Gcg-Cre [18], and POMC-Cre [41]. For the whole-brain mapping and analyses, GLP-1R-ires-Cre was crossed with Ai14 JaxMice (#007914), Rosa-CAG-LSL-tdTomato-WPRE::ΔNeo [36] to obtain GLP-1R-ires-Cre::Ai14 mice. In all cases, mice were randomized according to body weight in each experimental group. The investigators were blinded to the treatment. The sample size required n = 7–12 per group was based on the previous studies examining the effects of GLP-1 signaling on feeding behavior [32].

### Virus injection

The AAV virus (Adeno-Associated Virus) used include pAAV-EF1a-double floxed-hChR2 (H134R)-EYFP-WPRE-HGHpA (Catalog #20298-AAV1), pAAV-FLEX-tdTomato (Catalog #28305-AAV5) and pAAV-hSyn-DIO-hM3D (Gq)-mCherry (Catalog #44361-AAV9).

### Electrophysiology

Brain slice electrophysiology was conducted, as described elsewhere [20]. Mice were anesthetized, decapitated, and brains were removed and quickly immersed in cold (4 °C) oxygenated cutting solution containing (in mM): 50 sucrose, 2.5 KCl, 0.625 CaCl_2_, 1.2 MgCl_2_, 1.25 NaH2PO_4_, 25 NaHCO_3_, and 2.5 glucose. Coronal cerebral cortex slices, 300 mm in thickness, were cut using a vibratome (VT 1200S; Leica). Brain slices were collected in artificial cerebrospinal fluid (ACSF) and bubbled with 5%CO_2_ and 95%O_2_. The ACSF contained (in mM): 125 NaCl, 2.5 KCl, 2.5 CaCl_2_, 1.2 MgCl_2_, 1.25 NaH_2_PO_4_, 26 NaHCO_3_, and 2.5 glucose. After 1 h of recovery, slices were transferred to a recording chamber and constantly perfused with bath solution (30 °C) at a flow rate of 2 ml/min. Exn4 (10 nM) was added to the bath solution to stimulate the ARC GLP-1R neurons. Patch pipettes with a resistance of 8-10MΩ were made from borosilicate glass (World Precision Instruments) with a pipette puller (PC-10, Narishige) and filled with the pipette solution containing (in mM): 126 K-Gluconate, 4 KCl, 10 HEPES, 4 Mg-ATP, 0.3 Na_2_-GTP, 10 phosphocreatine (pH to 7.2 with KOH) for current and voltage-clamp recordings. All data were analyzed offline using ClampFit 10.2 (Molecular Devices, USA) software.

### Histology and immunohistochemistry assay

Mice were anesthetized with Euthasol and transcardially perfused with 4% PFA in PBS, pH 7.4. The brains were kept in 4% PFA overnight and then moved to 30% sucrose. Coronal brain slices (50 μm) were prepared thereafter. The numbers of GLP-1R-expressing (tdTomato) neurons in GLP-1R-ires-Cre::Ai14 mice were counted from a series of 50 μm sections (5 section intervals from section to section) using ImageJ. Images were acquired with a Zeiss LSM700 confocal microscope using a 546 nm laser. Z-stack images captured the entire thickness of the section at 10 µm steps for images taken with a 20× objective. Gcg-expressing neurons and their axon terminals were labeled with ChR2-eYFP by injecting AAV vectors in the Gcg-Cre mice. The semi-quantitation of the projections was based on the fluorescent signal area and intensity.

For the c-Fos staining, only slices with the ARC region were prepared. The standard IHC protocol was followed. The primary antibodies used were anti-c-Fos (1:1000, Santa Cruz, SC271243). AlexaFluor secondary antibodies (488-goat anti-rabbit, 1:500, Life Technologies) were used to visualize the signals, and images were acquired through confocal microscopy (Zeiss, LSM700).

c-Fos quantification: Total GLP-1R (RFP) and total c-Fos (Green) labeled cells were counted using the Cell Counter plugin in ImageJ. Then the overlapping signals between RFP and Green were measured as c-Fos + cells. This quantification was done for the control and target (hM3Dq) group, and the comparison was presented accordingly.

### Behavioral experiments

#### Chemogenetic activation of GLP-1R-expressing neurons 

Male mice were caged into groups of either 3 or 4. For the stimulation of the GLP-1R-expression neurons in the ARC, we injected 300 nL of pAAV-FLEX-tdTomato (Catalog #28305-AAV5) and pAAV-hSyn-DIO-hM3D (Gq)-mCherry (Catalog #44361-AAV9) in ARC at coordinates: Anteroposterior (AP): − 1.3 mm from bregma; mediolateral (ML): ± 0.2 mm, dorsovental (DV): − 6.2 to 6.1 mm. After four weeks of recovery, glucose tolerance tests (GTT) and food intake behavior experiments were conducted after an *i.p.* DREADD ligand injection.

#### Glucose tolerance test

The mice were fasted overnight (0.5 g chow was provided). The same protocol was used as described before [62]. At Time 0, blood glucose was measured to set a baseline. 1 mg/kg body weight CNO was injected intra-peritoneally. After 30 min, 1 g/kg body weight of 20% dextrose solution was injected i.p., and blood samples were collected from the tail vein at 15, 30, 60, and 120 min. For GTT, injection volume was calculated as mentioned below:

$${\text{Injection}}{\mkern 1mu} {\text{volume}}\left( {\mu {\mkern 1mu} {\text{L}}} \right) = {\text{mouse}}{\mkern 1mu} {\text{weight}}\left( {{\text{Kg}}} \right) \times \frac{{\frac{{2g}}{{kg}}glucose}}{{0.2\frac{g}{{ml}}}} = {\text{mouse}}{\mkern 1mu} {\text{weight}}\left( {{\text{kg}}} \right) \times 10\;{\text{ml/kg glucose}} = {\text{mouse}}{\mkern 1mu} {\text{weight}}({\text{g}}) \times 10$$

#### Food intake

Mice were singly housed before the experiment in a 12 h light/dark cycle with ad libitum access to water and were fasted overnight for 12 h (9 pm–9 am); access to only 0.5 g of food was given. The following day, each animal's weight was measured, CNO was prepared, and 1 mg/kg BW CNO was injected *i.p.* into each mouse. After 30 min, food was added to their cage. Standard chow intake was measured at t = 0,1,2,3,4,5 and 24 h. After the experiment, animals were grouped together into their respective cages.

#### Open field locomotor activity

Mice were single housed the night before the experiment. To test whether there are any side effects of the chemogenetic agent used, 1 mg/kg BW CNO was *i.p.* injected. After 30 min, mice were allowed to explore a novel environment for 6 min. They were put in the open field “exploratory stage,” and their activity and movement were recorded via the center cage software. These experiments were performed under dim light during the light cycle [63].

### Insulin measurements

After overnight fasting, CNO was injected as described before. After 120 min, Mice were anesthetized with Euthasol, and 1 ml of blood was collected from the heart and stored at − 80 °C. Plasma was separated by centrifugation at 6000 rpm for 10 min at 4 °C. Insulin levels were then analyzed using a mouse supersensitive insulin ELISA (ALPCO, Salem, NH, USA) [64].

### Quantification and statistical analysis

Statistical analysis was performed using Excel (version 2019). In addition, statistical analysis for blood glucose and food intake behavior was also analyzed using GraphPad Prism 8.0. All the data is presented as mean ± standard error of the mean (SEM). Data were analyzed using Student’s t-test and Two-way ANOVA using Geisser-Greenhouse’s epsilon correction. A p-value of less than 0.05 was considered statistically significant.

## Supplementary Information

**Additional file 1: Figure S1.** GLP-1 receptor neuron distribution in the whole brain. Representative images of GLP-1R-ires-cre mouse crossed with Ai14 tdTomato reporter mice line.

**Additional file 2: Figure S2.** GLP-1 receptor neuron distribution in the whole brain. Representative images of GLP-1R-ires-cre mouse crossed with Ai14 tdTomato reporter mice line.

**Additional file 3: Figure S3.** NTS GLP-1 neuron projection mapping in the whole brain. Representative images of Gcg-cre BAC mouse injected with DIO-Chr2-EYFP.

**Additional file 4: Figure S4.** NTS GLP-1 neuron projection mapping in the whole brain. Representative images of Gcg-cre BAC mouse injected with DIO-Chr2-EYFP.

## Data Availability

The data and datasets used and/or analyzed during the current study are available from the corresponding authors upon reasonable request.

## Abbreviations

AgRP: Agouti-related protein
ARC: The arcuate nucleus of the hypothalamus
CNO: Clozapine-N-oxide
CNS: Central nervous system
CRH: Corticotrophin-releasing hormone
DIO: Double inverted open reading frame
DR: Dorsal raphe nucleus
DREADDs: Designer Receptor Exclusively Activated by Designer Drugs
eYFP: Enhanced yellow fluorescent protein
Enx4: Exendin4
GFP: Green fluorescent protein
RFP: Red fluorescent protein
GLP-1: Glucagon-like peptide 1
GLP-1R: Glucagon-like peptide 1 receptor
GTT: Glucose tolerance test
LH: Lateral hypothalamus
MD: Medial dorsal nucleus
MnPO: Median preoptic nucleus
MvePC: Medial vestibular parvicellular subfields nucleus
NTS: Nucleus tractus solitarius
PnO: Pontine reticular nucleus, oral part
POMC: Pro-opiomelanocortin
PVN: Paraventricular hypothalamic nucleus
SFO: Subconical organ
SO: Supraoptic nucleus
VMH: Ventromedial nucleus of the hypothalamus
