# Supplementary figures and images for: Activation of arcuate nucleus glucagon-like peptide-1 receptor-expressing neurons suppresses food intake
Source: Cell Biosci. 2022 Oct 29;12:178. doi: 10.1186/s13578-022-00914-3 (PMC9618215; doi:10.1186/s13578-022-00914-3)

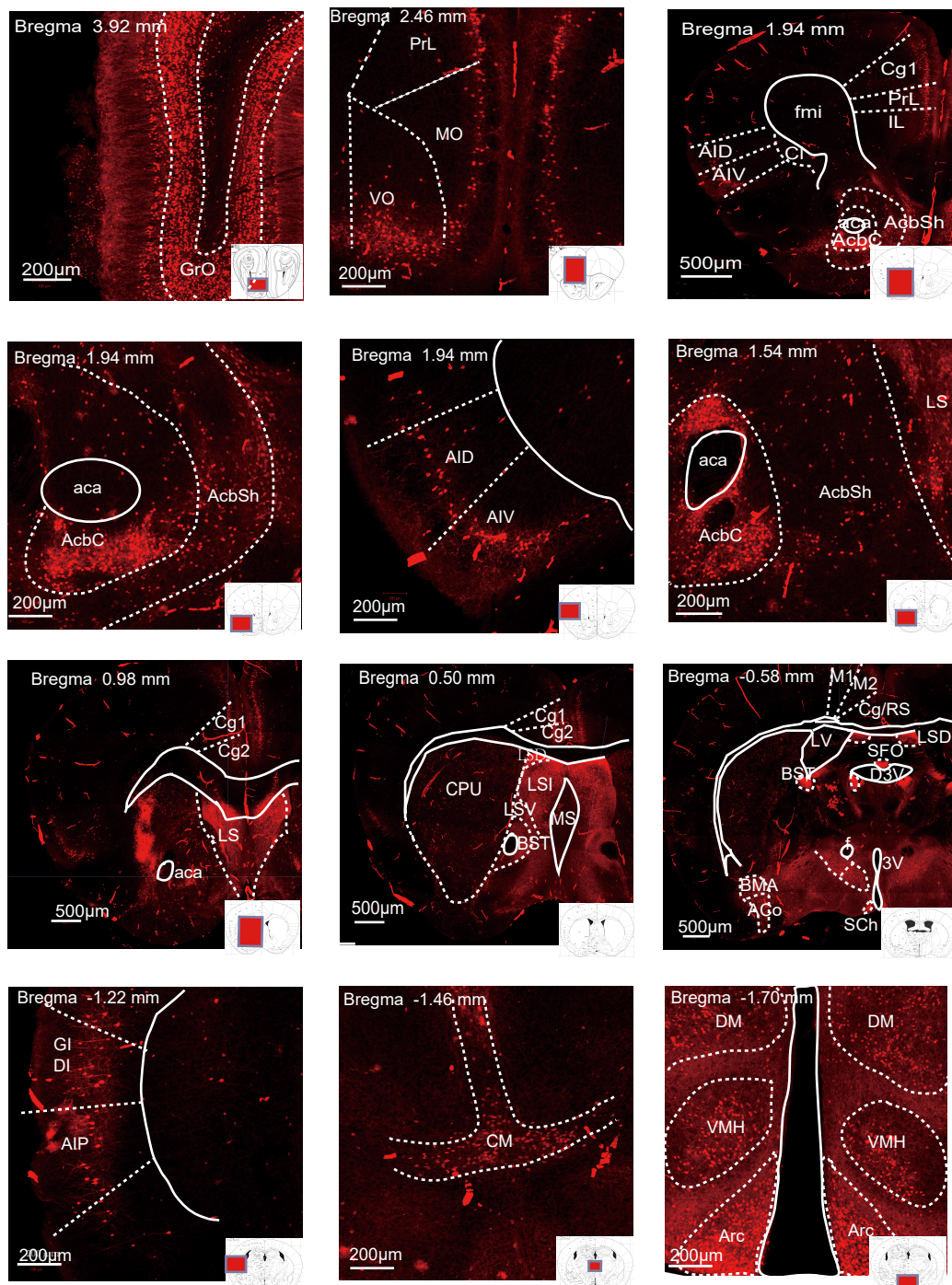

Singh et. al. Supplemental Figure 1

Supplement: Supplementary file 1 — Additional file 1: Figure S1. GLP-1 receptor neuron distribution in the whole brain. Representative images of GLP-1R-ires-cre mouse crossed with Ai14 tdTomato reporter mice line. [file 13578_2022_914_MOESM1_ESM.pdf]

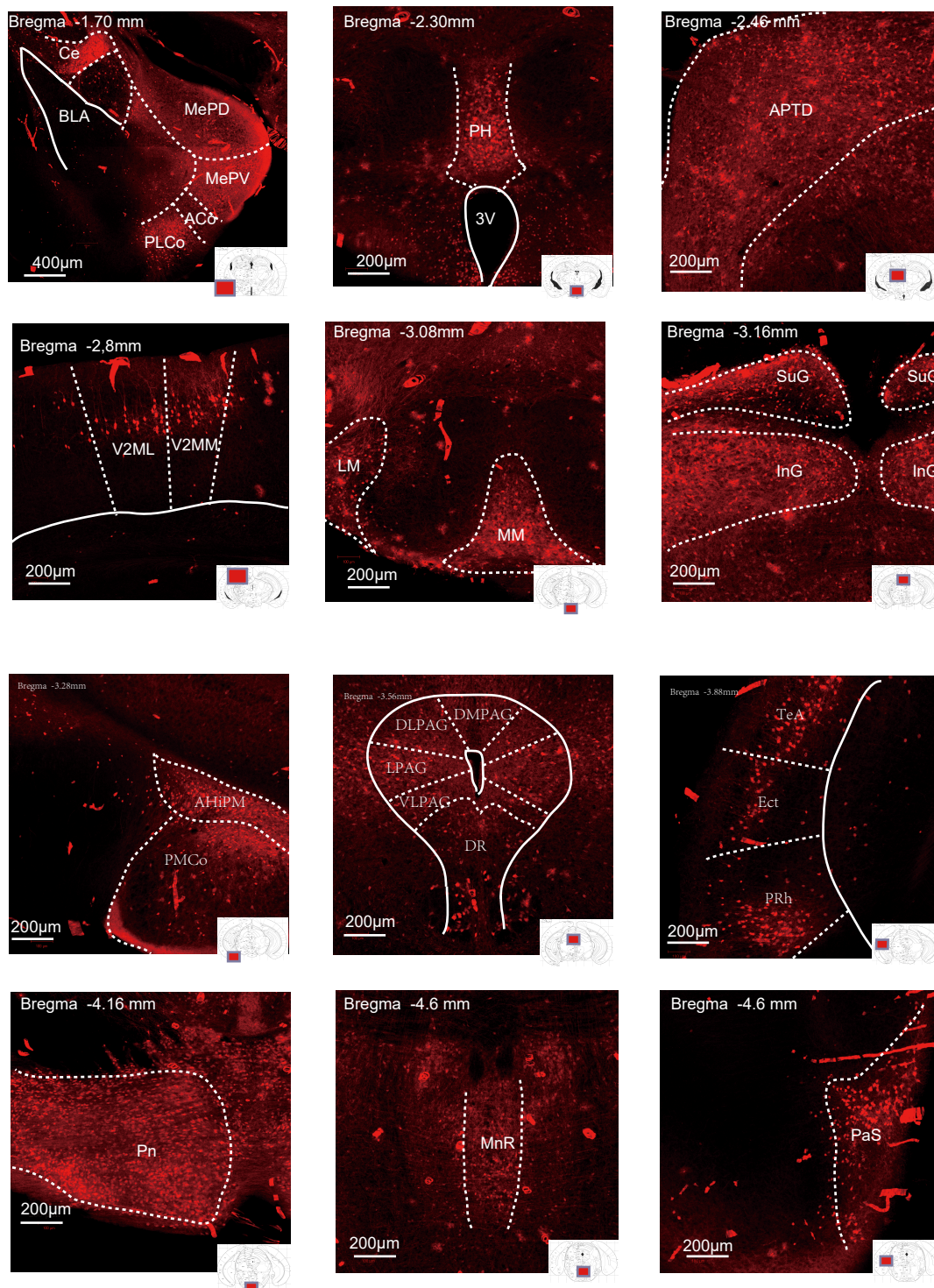

Singh et. al. Supplemental Figure 2

Supplement: Supplementary file 2 — Additional file 2: Figure S2. GLP-1 receptor neuron distribution in the whole brain. Representative images of GLP-1R-ires-cre mouse crossed with Ai14 tdTomato reporter mice line. [file 13578_2022_914_MOESM2_ESM.pdf]

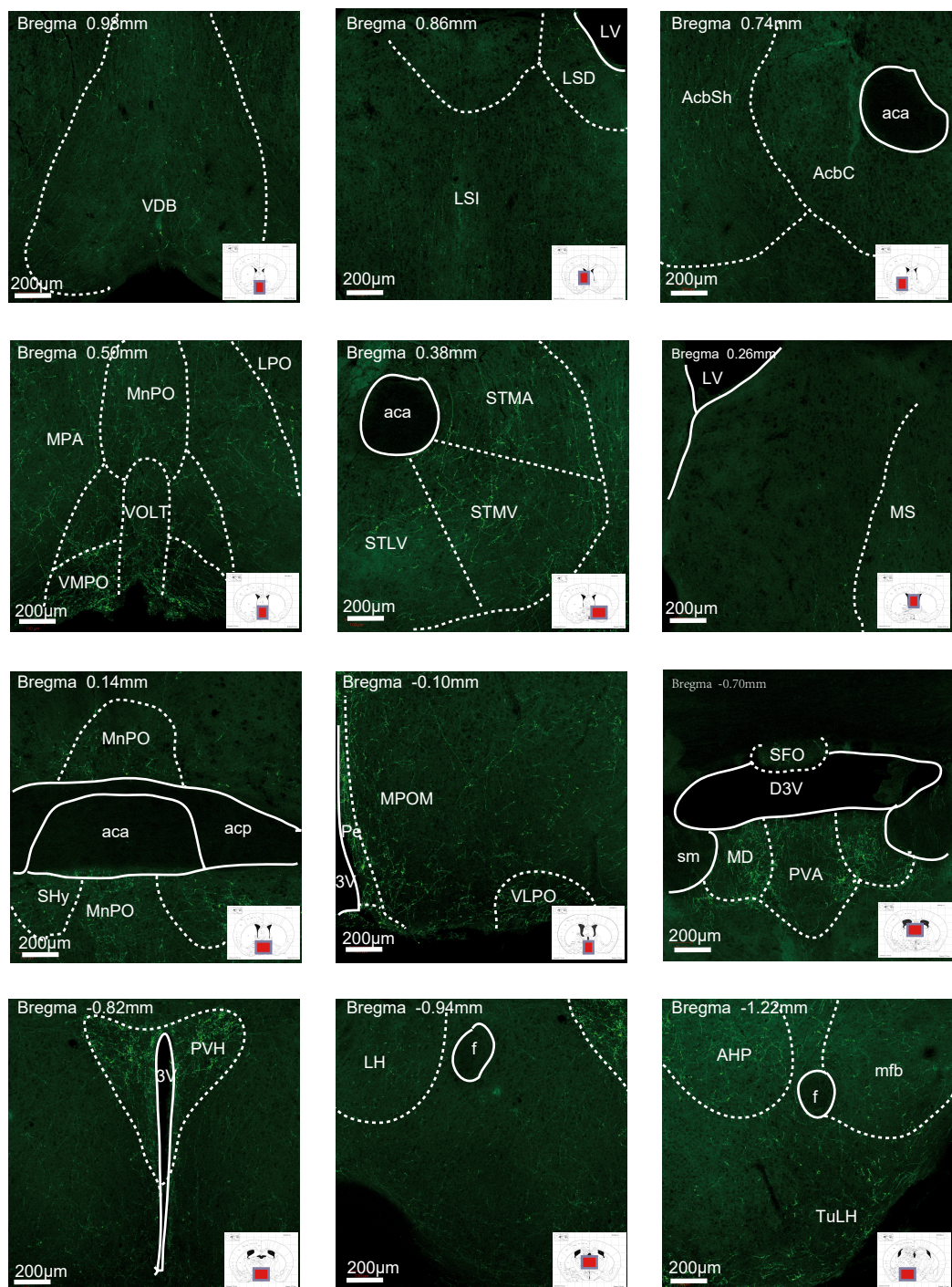

Singh et. al. Supplemental Figure 3

Supplement: Supplementary file 3 — Additional file 3: Figure S3. NTS GLP-1 neuron projection mapping in the whole brain. Representative images of Gcg-cre BAC mouse injected with DIO-Chr2-EYFP. [file 13578_2022_914_MOESM3_ESM.pdf]

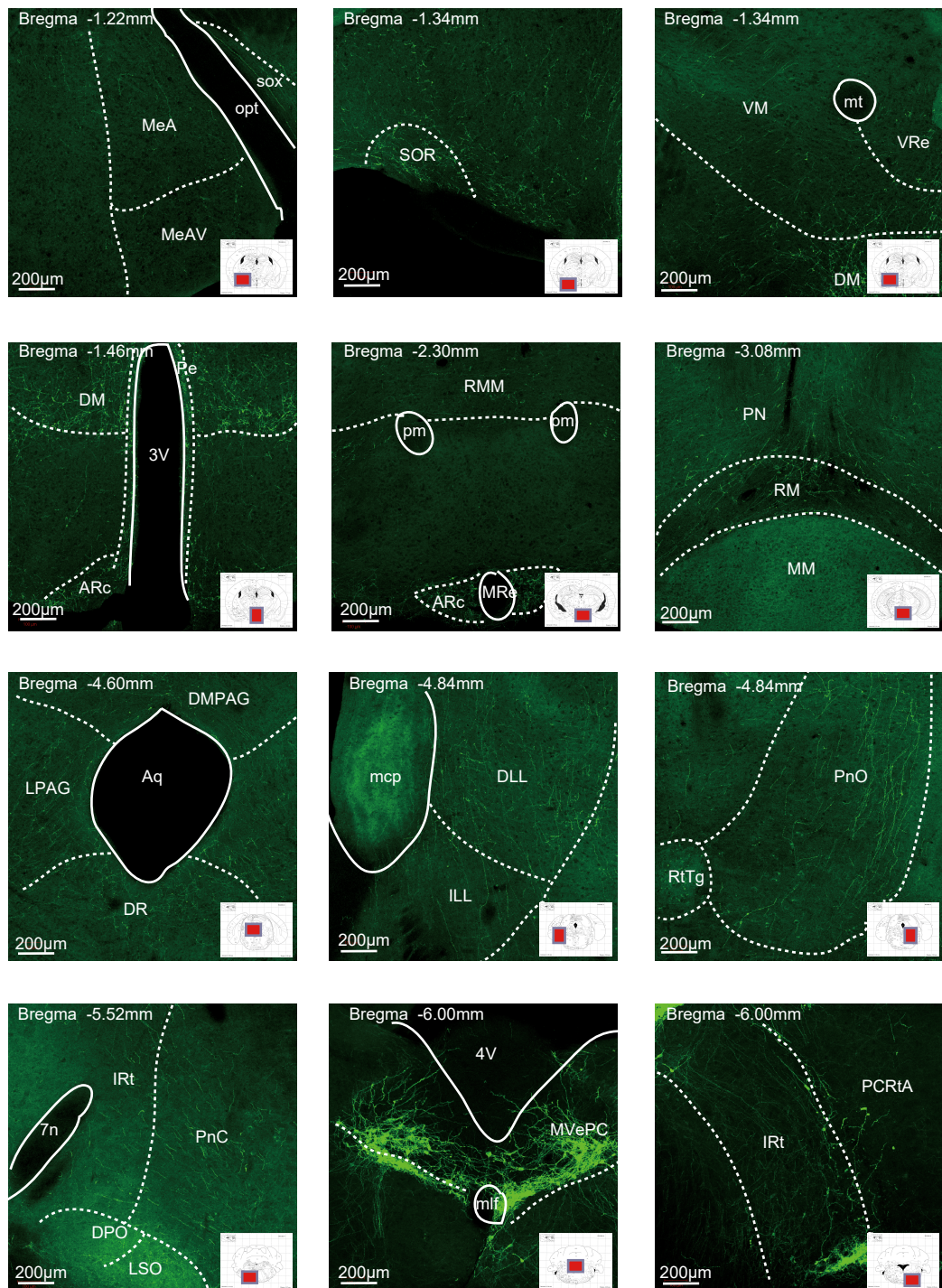

Singh et. al. Supplemental Figure 4

Supplement: Supplementary file 4 — Additional file 4: Figure S4. NTS GLP-1 neuron projection mapping in the whole brain. Representative images of Gcg-cre BAC mouse injected with DIO-Chr2-EYFP. [file 13578_2022_914_MOESM4_ESM.pdf]
